# Elemental Fingerprinting of Mussel Shells to Predict Population Sources and Redistribution Potential in the Gulf of Maine

**DOI:** 10.1371/journal.pone.0080868

**Published:** 2013-11-14

**Authors:** Cascade J. B. Sorte, Ron J. Etter, Robert Spackman, Elizabeth E. Boyle, Robyn E. Hannigan

**Affiliations:** 1 School for the Environment, University of Massachusetts, Boston, Massachusetts, United States of America; 2 Department of Biology, University of Massachusetts, Boston, Massachusetts, United States of America; 3 University of Great Falls, Great Falls, Montana, United States of America; Natural History Museum of Denmark, Denmark

## Abstract

As the climate warms, species that cannot tolerate changing conditions will only persist if they undergo range shifts. Redistribution ability may be particularly variable for benthic marine species that disperse as pelagic larvae in ocean currents. The blue mussel, *Mytilus edulis*, has recently experienced a warming-related range contraction in the southeastern USA and may face limitations to northward range shifts within the Gulf of Maine where dominant coastal currents flow southward. Thus, blue mussels might be especially vulnerable to warming, and understanding dispersal patterns is crucial given the species' relatively long planktonic larval period (>1 month). To determine whether trace elemental “fingerprints” incorporated in mussel shells could be used to identify population sources (i.e. collection locations), we assessed the geographic variation in shell chemistry of blue mussels collected from seven populations between Cape Cod, Massachusetts and northern Maine. Across this ∼500 km of coastline, we were able to successfully predict population sources for over two-thirds of juvenile individuals, with almost 80% of juveniles classified within one site of their collection location and 97% correctly classified to region. These results indicate that significant differences in elemental signatures of mussel shells exist between open-coast sites separated by ∼50 km throughout the Gulf of Maine. Our findings suggest that elemental “fingerprinting” is a promising approach for predicting redistribution potential of the blue mussel, an ecologically and economically important species in the region.

## Introduction

Recent increases in mean and extreme temperatures have been implicated in driving local populations and range-restricted species to extinction, and species extinctions may become increasingly common in the next century (see [1]–[3]). In order to persist (i.e., avoid extinction), species unable to tolerate changing climatic conditions must shift their ranges to track temperature isoclines [4]. Although poleward range shifts have already been documented for a number of species [5]–[7], redistribution ability is often unknown and unaccounted for in attempts to forecast species' ranges [8]. The challenge of assessing redistribution potential is particularly great in the case of benthic marine species for which the pelagic larval stage represents a “black box” in their complex life cycles [9]. Furthermore, populations inhabiting locations with currents that flow predominantly equatorward, opposite the direction of likely climate shifts, might face limitations to shifting poleward [10].

There is emerging evidence that global warming is precipitating declines in blue mussel, *Mytilus edulis*, populations of the eastern USA [11],[12]. Persistence of blue mussels in the Gulf of Maine may increasingly require northward range shifts, towards habitats that are cooler on average, as well as the “rescue” of more cold-adapted populations *via* re-seeding from more heat-tolerant populations [13],[14]. However, it is unknown whether mussels can disperse northward in the region against the predominantly southward flowing coastal currents [15]–[17]. Furthermore, the Gulf of Maine represents the center of the blue mussel's abundance distribution in the northwestern Atlantic, with current abundances 20-fold lower in southern Canada [18]. There would likely be severe ecological and economic impacts if blue mussel populations declined and, ultimately, if the species became unable to persist in the Gulf of Maine. As basal species, mussels serve as a primary food source for the top carnivores in intertidal habitats [19],[20] and are foundation species that, within the 3-dimensional matrix of their beds, provide essential habitat for a diverse assemblage of invertebrates [21]. Furthermore, blue mussels are consumed by humans, with over 6 million pounds – amounting to $7 million – harvested in the USA in 2011, and almost 90% of USA mussel landings on record coming from the Gulf of Maine [22].

Here, we assess whether geographic variation in the shell chemistry of blue mussels suggests that elemental fingerprinting would be an effective approach for identifying population sources and, thus, redistribution potential of this species in the northwestern Atlantic. The viability and efficacy of an elemental fingerprinting approach (*sensu* [23]) for “provenancing” (i.e., tracing individuals' geographic origins) can be age-, species-, and location-specific [24]–[27]. To determine whether the composition of trace metals, present in the water column and incorporated into mussel shells [28], is distinct between populations inhabiting the Gulf of Maine, we examined our ability to reclassify juvenile and adult mussels to known collection locations based on their geochemical signatures. Our results suggest that this approach could be used to better characterize connectivity patterns and persistence potential of blue mussels in the Gulf of Maine.

## Materials and Methods

**Figure 1:**
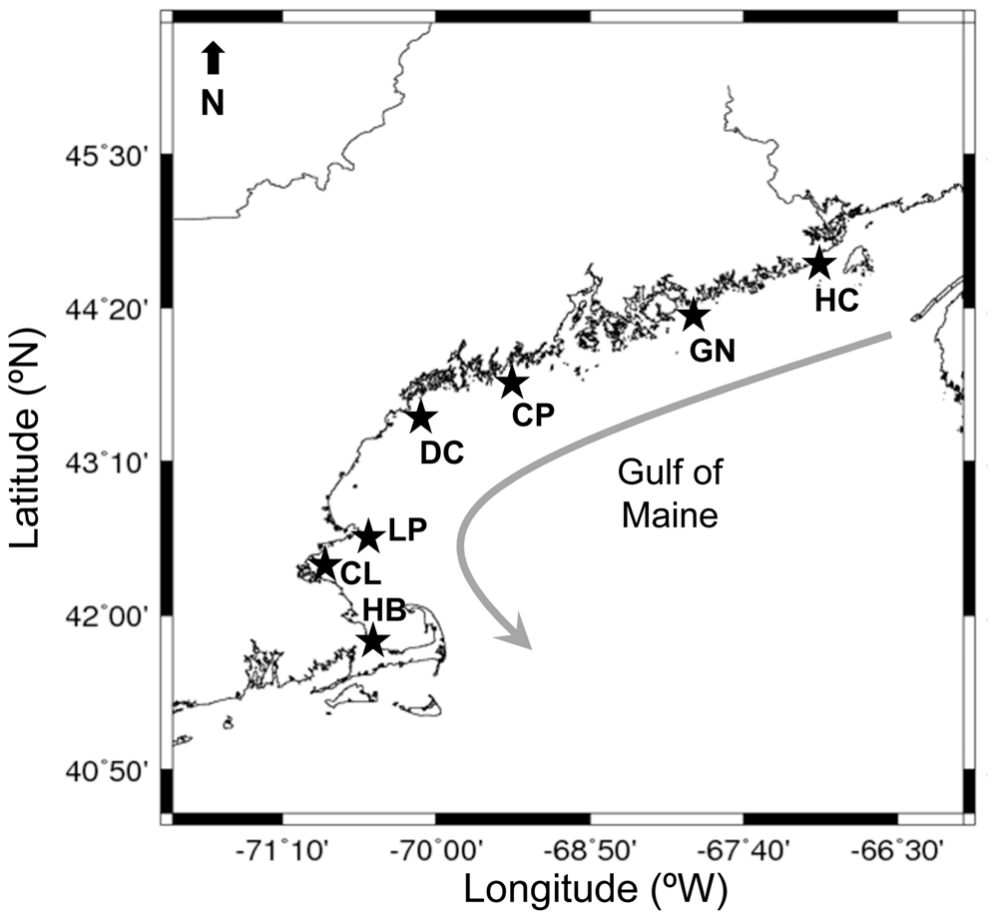
Locations of Gulf of Maine study sites. Sites include Hamilton Cove (HC), Grindstone Neck (GN), Chamberlain Point (CP), Dyers Cove (DC), Loblolly Point (LP), Cunner Ledge (CL), and Horizons Beach (HB). The gray arrow indicates the predominant current direction (see [17]).

To assess the reliability of elemental fingerprinting for identifying mussel source locations in the Gulf of Maine, we collected mussels at 7 sites between northern Maine and Cape Cod, Massachusetts, USA (Fig. 1). All sites supported mussel populations inhabiting coastal, intertidal, hard-bottom habitats that were on or adjacent to rocky headlands, with the exception of HB which was a rock jetty. No permits were required for the described study, which did not involve a protected species. Within a one-month period in early summer 2011, we collected *N* = 30 adult mussels (35.7±8.8 SD mm length) and *N* = 20–25 juvenile mussels (2.6±0.6 SD mm length) at each site, the juveniles being most often found attached to foliose algae or the byssal threads of adult mussels.

Mussels were transported to the laboratory on ice, where their soft tissues were removed. For shells from the northernmost site (HC; Fig. 1), where the congener *Mytilus trossulus* coexists – and is cryptogenic – with *M. edulis* at about 35% frequency [29], we saved the soft tissue and confirmed species identities using species-specific PCR markers for COI mtDNA (see Appendix S1; note that these congeners do not appear to hybridize in the Gulf of Maine, [18]). Shells were cleaned with a series of washes (1 min in glacial acetic acid and 2×1 min in ultrapure water). For adult shells, one valve was sectioned lengthwise using an IsoMet® diamond saw (Buehler, Illinois Toolworks Inc., Lake Bluff, Illinois, USA), and the thin section was mounted on a petrographic slide with Crystal Bond adhesive (Electron Microscopy Sciences, Hatfield, Pennsylvania, USA). Juvenile shells were small enough to be mounted whole, with the valves separated and exterior of the shells facing upwards away from the slides.

Using laser-ablation inductively-coupled mass spectrometry (LA-ICP-MS), we analyzed the trace elemental composition of shells at the growing edge (i.e., the most recently-formed shell) for both adults and juveniles. The LA-ICP-MS system consisted of a laser ablation unit (213 nm Nd:YAG; Cetac Technologies, Omaha, Nebraska, USA) connected to a Perkin Elmer ELAN DRC II ICP-MS (Perkin Elmer, Norwalk, Connecticut, USA). Laser ablation was conducted at 75% power with additional parameters as follows: for adults, we ablated three 50 µM spots (subsamples) with 300 shots at 10 Hz; for juveniles, we ablated a single line (parallel to shell growth lines) at 10 Hz with a spot size of 50 µM and a scan rate of 25 µM sec^−1^. We quantified isotopes of 8 trace elements for inclusion in our analyses (^26^Mg, ^46^Ca, ^63^Cu, ^66^Zn, ^86^Sr, ^137^Ba, ^139^La, and ^208^Pb) with GeoPro2010 software (Cetac Technologies). Using USGS standards (MACS-1 and MACS-3) and ^43^Ca as an internal standard, we calculated ratios to ^46^Ca for each of the trace elements.

We conducted a linear discriminant function analysis using Proc DISCRIM in SAS v. 9.2 (SAS Institute, Cary, North Carolina, USA) to assess our ability to distinguish between sites based on shell chemistry (by means of a jackknifed cross-validation analysis). Samples with values for any trace element below the instrument detection limit ([30]; 31/159 juveniles and 71/210 adults) were not included in the analysis; thus, we used proportional prior probabilities given unequal sample sizes. Results of the cross-validation analysis are only reported for individuals assigned to sites based on a posterior probability of greater than 0.4, approx. 3 times the random probability of 0.14. Individual elemental ratios were also compared between sites with univariate analyses of variance.

## Results

**Figure 2:**
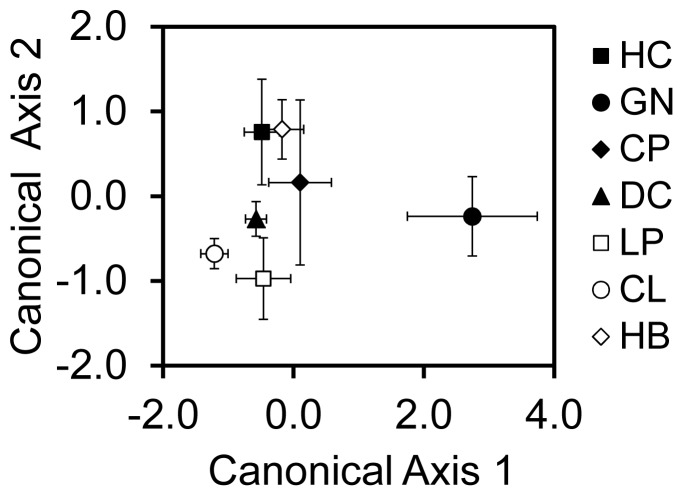
Canonical scores for discriminant function analysis of trace element concentrations in juvenile mussel shells. Values are site means (±95% confidence intervals). These two axes represent 79.3% of the variation between the seven collection locations in the Gulf of Maine, ordered in the legend from north (HC) to south (HB).

Using the cross-validation analysis, we were able to correctly predict the collection site for 68.4% of juvenile mussels (Table 1, Fig. 2), with 78.5% of individuals correctly classified to the site of collection or the adjacent site. Misclassifications were more frequent for the most open coast sites (HC, CP, LP) than for more sheltered sites. Grouping mussels into three regions – northern Maine (HC & GN), southern Maine (CP & DC), and Massachusetts (LP, CL & HB) – increased classification success to 97% (Table S1).

**Table 1:** Classification success of a linear discriminant function for juvenile mussels based on trace elemental composition of shell edges.

	Predicted site (columns)								
	HC	GN	CP	DC	LP	CL	HB	Total *N*	% correct
Collection site (rows)									
HC	8	0	0	2	3	0	3	16	50.0
GN	0	14	0	0	0	0	0	14	100.0
CP	1	1	2	3	0	1	1	9	22.2
DC	0	0	0	8	2	0	0	10	80.0
LP	0	0	0	2	4	0	0	6	66.7
CL	0	0	0	3	0	10	0	13	76.9
HB	1	0	0	2	0	0	8	11	72.7
Overall classification success									68.4%

Values are individual mussels from a known collection site (rows) classified (*via* jackknifed cross-validation, using each individual as a test case against a discriminant function based on the remaining mussels) into a predicted site (columns). Sites are listed in order from north (HC) to south (HB).

Classification success was higher for juveniles than for adults; 57.3% and 73.0% of adults were correctly classified to the collection site or within adjacent sites, respectively (Table S2). Classification success tended to increase with sample size, and individuals at sites with the highest levels of misclassification tended to classify into an adjacent site: for example, none of the adults from 2 sites (GN and DC) were re-assigned with high probability to their site of collection, but 4 of 5 individuals from GN and 6 of 10 individuals from DC were assigned to adjacent sites (Table S2).

**Figure 3:**
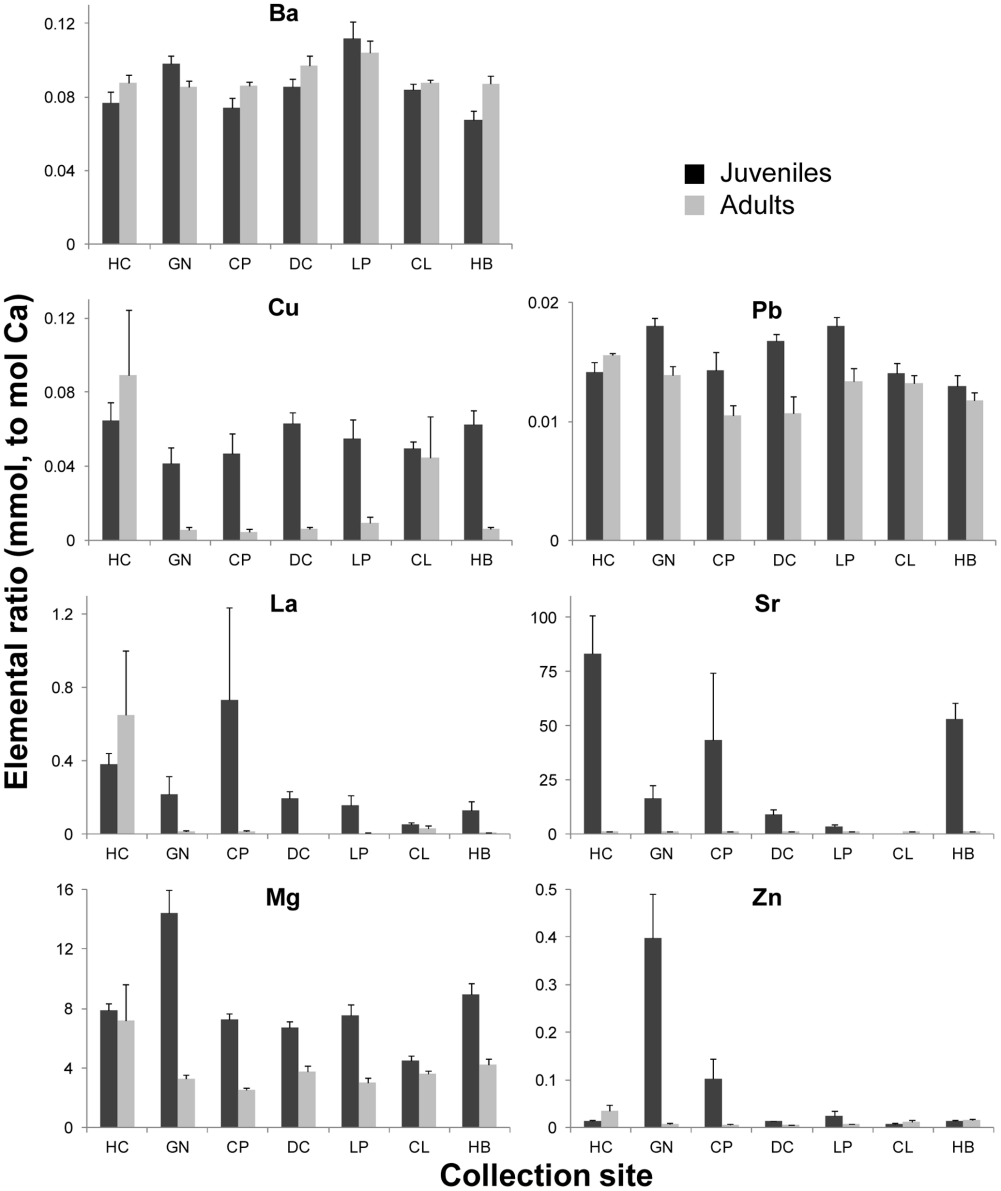
Trace elemental composition of mussel shells. Values are ratios (mmol, to mol Ca) of trace element concentrations (mean ± SE) in shells of juvenile (black) and adult mussels (gray) collected from 7 sites in the Gulf of Maine (see Fig. 1). Sites are listed in order from north (HC) to south (HB).

One-way ANOVA results for juvenile mussel shells indicated differences between sites for 5 of the elemental ratios (*p*<0.001), all except La:Ca (*p* = 0.0734) and Cu:Ca (*p* = 0.270) (Fig. 3). Of the 7 elemental ratios included in the linear discriminant function for juvenile shells, Mg:Ca, Zn:Ca, and Ba:Ca had the highest standardized coefficient values and were, thus, the most important for discriminating between sites (Table S3). For adult mussel shells, all 7 elemental ratios differed across sites (*p*<0.04), with Sr:Ca, Ba:Ca, and Zn:Ca the most important ratios for differentiating between sites.

## Discussion

Our results show that trace element fingerprinting based on shell chemistry can be used to successfully identify the population source for blue mussels in the Gulf of Maine, an advective, open-coastal system. Over two-thirds (68%) of mussels were correctly classified to their site of collection with almost 80% classified no more than one site distant from their collection site. These results are slightly higher than those for the congeners *Mytilus californianus* and *M. galloprovincialis* in southern California, USA where 56% were correctly classified to one of six collection sites spanning approx. 50 km of coastline [23]. As found by Becker et al. [23], for which grouping California sites into two (northern and southern) regions increased classification ability, our overall classification success was 97% when sites were grouped into regions of northern Maine, southern Maine, and Massachusetts.

Ultimately, for predicting origins of mussel larvae, it would be ideal to maintain separate site identities because calculations based on current speeds (5–30 cm sec^−1^; [17]) and *M. edulis* larval duration (≥1 month; [31]) suggest dispersal could commonly occur between sites but within a region in the Gulf of Maine. Clear site-specific elemental signatures allowed us to assign sources with 66–100% accuracy at five of our seven sites. These site signatures represent an amalgamation of the 7 elements, most of which varied significantly along the coastline. Levels of heavy metals in mussel soft tissues, monitored by the Mussel Watch program, have also demonstrated high site-to-site variability, with high heavy metal concentrations associated with low tidal ranges (i.e. low flushing rate) and high population sizes [32]. Consequently, soft tissue levels of some heavy metals (e.g. Pb) tend to increase towards the south [32], a pattern that is not apparent across our smaller sample of 7 sites. In our study, the GN site, in particular, stands out as having high levels of Mg and Zn; the latter may be a remnant of historical mining activities as elevated Zn levels were recently detected in estuarine sediments of the watershed inshore from GN [33]. Two sites (HC and CP) had much lower assignment accuracy. Although the reason for this significantly reduced accuracy is unclear, localized signatures may be obscured at these sites due to their location on more open coasts with greater rates of water exchange. Open coasts tend to have geochemical signatures that differ on a larger scale (e.g., [26],[34]) relative to those in bays or estuaries where water exchange is less (e.g., [35],[36]).

Several methods have been employed for predicting dispersal trajectories and connectivity patterns of marine species with pelagic larvae, including elemental fingerprinting, genetic techniques, bio-physical modeling, and individual-based biological models of larval development (reviewed in [37]–[40]). Each of these approaches has different advantages and disadvantages such that a combined approach will often provide the greatest insight [40]. Trace element fingerprinting is an individual (rather than a population) metric that does not require assumptions about the dispersal pathway; rather, for each individual, the endpoint is known and starting point is determined based on many possible origins. Elemental fingerprinting typically incorporates several assumptions that are inherent to most assignment methods, including, for example, that the reference map from which larval origins are determined includes all possibilities, an assumption that is violated if there are populations outside of the study region whose larvae are transported into the study region. Such violations might be particularly problematic for predicting the origin of recruits on the edge of the study area, manifesting as high-levels of self-recruitment or misidentification of the natal site to one within the study region that is most similar to the uncharacterized site outside of the study region. Furthermore, the larval fingerprinting technique assumes that all locations within the study region are adequately characterized by the closest sampled site. Finally, elemental fingerprinting techniques are more reliable when there is greater site-specific consistency in dissolved concentrations of trace metals. Unsurprisingly, we found that classification success was higher with juvenile mussels than for adults, indicating that future attempts to identify larval origins and mussel connectivity patterns are likely to be more successful when using juvenile than adult shells. This was expected given that the same total shell volume was sampled for both ages, and it represented integration of trace elements incorporated over a shorter time period (and, thus, less potential temporal variation in water chemistry) in the juveniles, due to their faster growth rates as compared to adult mussels [41].

Keeping these caveats in mind, our results suggest that mussel population sources can be successfully identified in the Gulf of Maine, an advective, open-coast system. Trace element fingerprinting is, thus, a promising approach for predicting the larval origins of mussel recruits in the northeastern USA. Our preliminary analyses of larval shells (which are retained – and were sampled – at the umbo of juvenile shells) across these 7 sites suggest that blue mussels are able to disperse northward in the Gulf of Maine (Sorte et al. unpublished data). This pattern begs sampling with increased spatial and temporal coverage, as well as consideration of findings that trace metal incorporation can differ between larval and juvenile bivalves [42]. However, if our preliminary results suggesting northward dispersal in the Gulf of Maine are upheld by future research, they have positive implications for mussel persistence and redistribution potential in the region. The explanations for dispersal upstream (in relation to average currents) are the same as solutions to the “drift paradox”, a term that describes the difficulty of persistence for populations of especially benthic species maintaining their existing ranges in advective environments [43]. Pachepsky et al. [44] and Lutscher et al. [45] have shown that such maintenance of present range boundaries is theoretically synonymous with ability of a species to shift its range upstream. Byers and Pringle [46] addressed the conditions that might allow benthic marine invertebrates to disperse “against the flow” as pelagic larvae. Their modeling results indicated that temporal and spatial flow variation coupled with life-history characteristics (such as timing of reproduction and pelagic larval duration) can promote upstream dispersal. In a test of the “drift paradox” with the congeners *Mytilus californianus* and *M. galloprovincialis*, Carson et al. [47] found that self-recruitment was the dominant mechanism of persistence for upstream, range-edge populations in the eastern Pacific, and local retention is likely promoted by flow variability in our study region. Although measurements of both Eulerian flow (*via* fixed buoys) and Lagrangian flow (*via* drifters) support a predominantly southward directionality of coastal currents in the Gulf of Maine, there is spatial and temporal variation in the flow field that includes frequent northward excursions [15],[17],[48]. Additional evidence for conditions allowing some upstream dispersal in the Gulf of Maine comes from another benthic invertebrate: the green crab, *Carcinus maenas*. In this case, although genetic results suggest primarily downstream dispersal, the appearance of a southern allele in northern green crab populations indicates the presence of northward gene flow [49].

In conclusion, we have shown that significant differences in elemental signatures of mussel shells exist on 50 km scales throughout the Gulf of Maine, which should allow us to use elemental fingerprinting to identify natal origins and estimate patterns of blue mussel dispersal in the region. Although, to date, researchers are still in the process of confirming the viability of this “new wave” technique [50] of elemental fingerprinting (e.g., [26],[27],[35],[36],[51]), it has the potential to permit testing of important questions in global change biology, including whether oceanographic processes will preclude or facilitate climate-induced shifts in distributions of marine species. Ideally, future use of elemental fingerprinting for characterizing mussel dispersal patterns would maximize study area, coverage of that area (i.e., number of collection sites), and sampling frequency while also integrating genetic and/or flow dynamic approaches to best shed light on the “black box” of larval connectivity. If future research, building off of the approach proposed here, indicates that blue mussels are indeed able to disperse northward in the Gulf of Maine, this suggests a natural mechanism that could allow populations to persist in the region, albeit increasingly shifted toward more northern locations.

## Supporting Information

Table S1 **Classification success (by region) of a linear discriminant function for juvenile mussel shells based on trace elemental composition.**
(PDF)

Table S2 **Classification success of a linear discriminant function for adult mussel shells based on trace elemental composition.**
(PDF)

Table S3 **Standardized canonical coefficients for the linear discriminant function based on shell chemistry of mussel juveniles.**
(PDF)

Appendix S1 **Supplementary methods for identification of** ***Mytilus*** **congeners from the northernmost HC site using species-specific PCR markers for the female lineage of COI mtDNA.**
(PDF)

## References

[1] Easterling DR, Meehl GA, Parmesan C, Changnon SA, Karl TR, et al (2000) Climate extremes: observations, modeling, and impacts. Science 289: 2068–2074. 11000103 10.1126/science.289.5487.2068

[2] Parmesan C (2006) Ecological and evolutionary responses to recent climate change. Annu Rev Ecol Evol Syst 37: 637–669.

[3] Meehl GA, Stocker TF, Collins WD, Friedlingstein P, Gaye AT, et al. (2007) Global climate projections. In: Solomon S, Qin D, Manning M, Chen Z, Marquis M, et al.. editors. Climate Change 2007: The Physical Science Basis. Contribution of Working Group I to the Fourth Assessment Report of the Intergovernmental Panel on Climate Change. Cambridge: Cambridge University Press.

[4] Berg MP, Kiers ET, Driessen G, van der Heijden M, Kooi BW, et al (2010) Adapt or disperse: understanding species persistence in a changing world. Glob Change Biol 16: 587–598.

[5] Parmesan C, Yohe G (2003) A globally coherent fingerprint of climate change impacts across natural systems. Nature 421: 37–42. 12511946 10.1038/nature01286

[6] Root TL, Price JT, Hall KR, Schneider SH, Rosenzweig C, et al (2003) Fingerprints of global warming on wild animals and plants. Nature 421: 57–60. 12511952 10.1038/nature01333

[7] Sorte CJB, Williams SL, Carlton JT (2010) Marine range shifts and species introductions: comparative spread rates and community impacts. Glob Ecol Biogeogr 19: 303–316.

[8] Pearson RG, Dawson TP (2003) Predicting the impacts of climate change on the distribution of species: are bioclimate envelope models useful? Glob Ecol Biogeogr 12: 361–371.

[9] Pineda J, Hare JA, Sponaugle S (2007) Larval transport and dispersal in the coastal ocean and consequences for population connectivity. Oceanogr 20: 22–39.

[10] Sorte CJB (2013) Predicting persistence in a changing climate: flow direction and limitations to redistribution. Oikos 122: 161–170.

[11] Jones SJ, Mieszkowska N, Wethey DS (2009) Linking thermal tolerances and biogeography: *Mytilus edulis* (L.) at its southern limit on the east coast of the United States. Biol Bull 217: 73–85. 19679724 10.1086/BBLv217n1p73

[12] Jones SJ, Lima FP, Wethey DS (2010) Rising environmental temperatures and biogeography: poleward range contraction of the blue mussel, *Mytilus edulis* L., in the western Atlantic. J Biogeogr 37: 2243–2259.

[13] Brown JH, Kodric-Brown A (1977) Turnover rates in insular biogeography: effect of immigration on extinction. Ecology 58: 445–449.

[14] Sorte CJB, Jones SJ, Miller LP (2011) Geographic variation in temperature tolerance as an indicator of potential population responses to climate change. J Exp Mar Biol Ecol 400: 209–217.

[15] Lynch DR, Ip JTC, Naimie CE, Werner FE (1996) Comprehensive coastal circulation model with application to the Gulf of Maine. Cont Shelf Res 16: 875–906.

[16] Anderson DM (1997) Bloom dynamics of toxic *Alexandrium* species in the northeastern U.S. Limnol Oceanogr. 42: 1009–1022.

[17] Pettigrew NR, Churchill JH, Janzen CD, Mangum LJ, Signell RP, et al (2005) The kinematic and hydographic structure of the Gulf of Maine Coastal Current. Deep-Sea Res 52: 2369–2391.

[18] Tam JC, Scrosati RA (2011) Mussel and dogwhelk distribution along the north-west Atlantic coast: testing predictions derived from the abundant-centre model. J Biogeogr 38: 1536–1545.

[19] Paine RT (1966) Food web complexity and species diversity. Am Nat 100: 65–75.

[20] Menge BA (1976) Organization of the New England rocky intertidal community: role of predation, competition, and environmental heterogeneity. Ecol Monogr 46: 355–393.

[21] Suchanek TH (1987) Extreme biodiversity in the marine environment: mussel bed communities of *Mytilus californianus* . Northwest Environ J 8: 150–152.

[22] National Oceanic and Atmospheric Association (2013) NOAA Fisheries Landings. Available: www.st.nmfs.noaa.gov/st1//commercial/landings/annual_landings.html. Accessed 08 January 2013.

[23] Becker BJ, Fodrie FJ, McMillan PA, Levin LA (2005) Spatial and temporal variation in trace elemental fingerprints of mytilid mussel shells: a precursor to invertebrate larval tracking. Limnol Oceanogr 50: 48–61.

[24] Becker BJ, Levin LA, Fodrie FJ, McMillan PA (2007) Complex larval connectivity patterns among marine invertebrate populations. Proc Natl Acad Sci USA 104: 3267–3272. 17360636 10.1073/pnas.0611651104 PMC1802003

[25] Fodrie FJ, Becker BJ, Levin LA, Gruenthal K, McMillan PA (2011) Connectivity clues from short-term variability in settlement and geochemical tags of mytilid mussels. J Sea Res 65: 141–150.

[26] Carson HS, López-Duarte PC, Cook GS, Fodrie FJ, Becker BJ, et al (2013) Temporal, spatial, and interspecific variation in geochemical signatures within fish otoliths, bivalve larval shells, and crustacean larvae. Mar Ecol Prog Ser 473: 133–148.

[27] Miller SH, Morgan SG, White JW, Green PG (2013) Interannual variability in an atlas of trace element signatures for determining population connectivity. Mar Ecol Prog Ser 474: 179–190.

[28] Thorrold SR, Jones GP, Hellberg ME, Burton RS, Swearer SE, et al (2002) Quantifying larval retention and connectivity in marine populations with artificial and natural markers. Bull Mar Sci 70: 291–308.

[29] Hayhurst S, Rawson PD (2009) Species-specific variation in larval survival and patterns of distribution for the blue mussels *Mytilus edulis* and *Mytilus trossulus* in the Gulf of Maine. J Moll Stud 75: 215–222.

[30] PerkinElmer Life and Analytical Sciences (2004) ELAN DRC II. Available: http://www.esc.cam.ac.uk/esc/files/Department/facilities/icp-ms/drcii-b.pdf. Accessed 09 October 2013.

[31] Seed R (1969) The ecology of *Mytilus edulis* L. (Lamellibranchiata) on exposed rocky shores. I. Breeding and settlement. Oecologia 3: 277–316. 28308905 10.1007/BF00390380

[32] Chase ME, Jones SH, Hennigar P, Sowles J, Harding GCH, et al (2001) Gulfwatch: Monitoring spatial and temporal patterns of trace metal and organic contaminants in the Gulf of Maine (1991–1997) with the blue mussel, *Mytilus edulis* L. Mar Poll Bull. 42: 491–505. 10.1016/s0025-326x(00)00193-4 11468927

[33] Osher LJ, Leclerc L, Wiersma GB, Hess CT, Guiseppe VE (2006) Heavy metal contamination from historical mining in upland soil and estuarine sediments of Egypt Bay, Maine, USA. Estuar Coast Shelf Sci 70: 169–179.

[34] López-Duarte PC, Carson HC, Cook GS, Fodrie FJ, Becker BJ, et al (2012) What controls connectivity? An empirical, multi-species approach. Integr Compar Biol 52: 511–524. 10.1093/icb/ics104 22888173

[35] Broadaway BJ, Hannigan RE (2012) Elemental fingerprints used to identify essential habitats: Nantucket Bay Scallop. J Shellfish Res 31: 671–676.

[36] Cathey AM, Miller NR, Kimmel DG (2012) Microchemistry of juvenile *Mercenaria mercenaria* shell: implications for modeling larval dispersal. Mar Ecol Prog Ser 465: 155–168.

[37] Levin LA (2006) Recent progress in understanding larval dispersal: new directions and digressions. Integr Compar Biol 46: 282–297. 10.1093/icb/icj024 21672742

[38] Thorrold SR, Zacherl DC, Levin LA (2007) Population connectivity and larval dispersal: using geochemical signatures in calcified structures. Oceanogr 20: 80–89.

[39] Cowen RK, Sponaugle S (2009) Larval dispersal and marine population connectivity. Annu Rev Mar Sci 1: 443–466. 10.1146/annurev.marine.010908.163757 21141044

[40] Leis JM, van Herwerden L, Patterson H (2011) Estimating connectivity in marine fish populations: What works best? Oceanogr Mar Biol 49: 205–246.

[41] Sukhotin AA, Abele D, Pörtner H-O (2002) Growth, metabolism and lipid peroxidation in *Mytilus edulis*: age and size effects. Mar Ecol Prog Ser 226: 223–234.

[42] Strasser CA, Mullineaux LS, Walther BD (2008) Growth rate and age effects on *Mya arenaria* shell chemistry: Implications for biogeochemical studies. J Exp Mar Biol Ecol 355: 153–163.

[43] Hershey AE, Pastor J, Peterson BJ, Kling GW (1993) Stable isotopes resolve the drift paradox for *Baetis* mayflies in an arctic river. Ecology 74: 2315–2325.

[44] Pachepsky E, Lutscher F, Nisbet RM, Lewis MA (2005) Persistence, spread and the drift paradox. Theor Popul Biol 67: 61–73. 15649524 10.1016/j.tpb.2004.09.001

[45] Lutscher F, Nisbet RM, Pachepsky E (2010) Population persistence in the face of advection. Theor Ecol 3: 271–284.

[46] Byers JE, Pringle JM (2006) Going against the flow: retention, range limits and invasions in advective environments. Mar Ecol Prog Ser 313: 27–41.

[47] Carson HS, Cook GS, López-Duarte PC, Levin LA (2011) Evaluating the importance of demographic connectivity in a marine metapopulation. Ecology 92: 1972–1984. 22073788 10.1890/11-0488.1

[48] Pringle JM (2006) Sources of variability in Gulf of Maine circulation, and the observations needed to model it. Deep-Sea Res II 53: 2457–2476.

[49] Pringle JM, Blakeslee AMH, Byers JE, Roman J (2011) Asymmetric dispersal allows an upstream region to control population structure throughout a species' range. Proc Natl Acad Sci USA 108: 15288–15293. 21876126 10.1073/pnas.1100473108 PMC3174593

[50] Palumbi SR, Gaines SD, Leslie H, Warner RR (2003) New wave: high-tech tools to help marine reserve research. Front Ecol Environ 1: 73–79.

[51] Bradbury IR, DiBacco C, Thorrold SR, Snelgrove PVR, Campana SE (2011) Resolving natal tags using otolith geochemistry in an estuarine fish, rainbow smelt *Osmerus mordax* . Mar Ecol Prog Ser 433: 195–204.

