# Supplementary material for: Elemental Fingerprinting of Mussel Shells to Predict Population Sources and Redistribution Potential in the Gulf of Maine
Source: PLoS One. 2013 Nov 14;8(11):e80868. doi: 10.1371/journal.pone.0080868 (PMC3828252; doi:10.1371/journal.pone.0080868)
Supplement: Table S1 — Classification success (by region) of a linear discriminant function for juvenile mussel shells based on trace elemental composition. (PDF) [file pone.0080868.s001.pdf]

Table S1. Classification success (by region) of a linear discriminant function for juvenile mussel shells based on trace elemental composition.

	Predicted region (columns)				
	No. ME	So. ME	MA	Total *N*	% correct
Collection region (rows)					
No. ME	23	0	0	23	100.0
So. ME	0	11	0	11	100.0
MA	0	2	33	35	94.3
Overall classification success					97.1%

Values are individual mussels from a known collection region (rows) classified (*via* jackknifed cross-validation, using each individual as a test case against a discriminant function based on the remaining mussels) into a predicted region (columns), either northern Maine (HC & GN), southern Maine (CP & DC), or Massachusetts (LP, CL & HB; see Fig. 1 for sites).
