# Supplementary material for: Elemental Fingerprinting of Mussel Shells to Predict Population Sources and Redistribution Potential in the Gulf of Maine
Source: PLoS One. 2013 Nov 14;8(11):e80868. doi: 10.1371/journal.pone.0080868 (PMC3828252; doi:10.1371/journal.pone.0080868)
Supplement: Table S2 — Classification success of a linear discriminant function for adult mussel shells based on trace elemental composition. (PDF) [file pone.0080868.s002.pdf]

Table S2. Classification success of a linear discriminant function for adult mussel shells based on trace elemental composition.

	Predicted site (columns)								
	HC	GN	CP	DC	LP	CL	HB	Total *N*	% correct
Collection site (rows)									
HC	6	0	0	0	3	6	2	17	35.3
GN	0	0	4	0	0	1	0	5	0.0
CP	0	2	18	0	1	0	0	21	85.7
DC	0	2	6	0	0	2	0	10	0.0
LP	0	0	2	0	7	0	0	9	77.8
CL	3	0	1	0	0	5	0	9	55.6
HB	0	0	0	1	0	2	15	18	83.3
Overall classification success									57.3%

Values are individual mussels from a known collection site (rows) classified (*via* jackknifed cross-validation, using each individual as a test case against a discriminant function based on the remaining mussels) into a predicted site (columns). Sites are listed in order from north (HC) to south (HB).
