# Supplementary material for: Elemental Fingerprinting of Mussel Shells to Predict Population Sources and Redistribution Potential in the Gulf of Maine
Source: PLoS One. 2013 Nov 14;8(11):e80868. doi: 10.1371/journal.pone.0080868 (PMC3828252; doi:10.1371/journal.pone.0080868)
Supplement: Table S3 — Standardized canonical coefficients for the linear discriminant function based on shell chemistry of mussel juveniles. (PDF) [file pone.0080868.s003.pdf]

Table S3. Standardized canonical coefficients for the linear discriminant function based on shell chemistry of mussel juveniles.

	Can1	Can2	Can3	Can4	Can5	Can6
Element (ratio to Ca)						
Ba	-0.3283	-0.7222	1.1749	0.2322	-0.9400	-0.1508
Cu	-0.5084	0.0687	-0.1963	0.5667	0.0909	0.4903
La	0.2014	-0.0659	0.4281	-0.9489	0.2715	-0.4204
Mg	1.1246	0.4654	-0.4164	0.4245	0.0394	-0.7617
Pb	0.3624	0.1987	0.1469	0.1565	1.3597	0.2836
Sr	-0.0923	0.6561	1.1235	0.1952	-0.1203	0.3868
Zn	0.6370	-0.2970	0.0076	-0.3748	-0.3180	0.8970

Coefficients are given for the elemental ratios (mmol, to mol Ca) and are representative of each element's importance in discriminating between collection sites.
