# Supplementary material for: Elemental Fingerprinting of Mussel Shells to Predict Population Sources and Redistribution Potential in the Gulf of Maine
Source: PLoS One. 2013 Nov 14;8(11):e80868. doi: 10.1371/journal.pone.0080868 (PMC3828252; doi:10.1371/journal.pone.0080868)
Supplement: Appendix S1 — Supplementary methods for identification of Mytilus congeners from the northernmost HC site using species-specific PCR markers for the female lineage of COI mtDNA. (PDF) [file pone.0080868.s004.pdf]

Appendix S1. Supplementary methods for identification of *Mytilus* congeners from the northernmost HC site using species-specific PCR markers for the female lineage of COI mtDNA.

Supplementary methods

To identify whether specimens were *Mytilus edulis* or *M. trossulus,* DNA was extracted using a Qiagen mini kit following the standard protocol. DNA was amplified from the female mitochondrial Cytochrome oxidase 1 (COI) gene using a PCR protocol developed by Paul Rawson. The following primers were used: CO1Ed1R (CACCTACCCCTTTATCCGTT); CO1Ed1L (TTTGTTCGGGGCAAGGT); CO1MTR2R CCGTTCTAAAGGACAGTATTAG); CO1MT2L GCAAGGTTGAGGTTAATGAT. PCR was conducted in a 25 µL reaction volume consisting of 1µL DNA, 2.5 µL Promega Go Taq flexi buffer, 1.5 mM MgCl2, 15uM each primer, 10mM dNTPs, 0.2 uL Promega hot start TAQ and water. PCR conditions were an initial 2 min denaturation at 94ºC followed by 30 cycles of 94ºC for 30 sec, 50ºC for 30 sec, 72ºC for 3 min, and a final extension of 5 min at 72ºC. 12uL of PCR product was run out in 4% agarose gel at 75 volts for 120 min. Products from *Mytilus trossulus* were 260 bp; those from *M. edulis* were 280 bp. All PCR reactions contained known *Mytilus* standards previously identified by P. Rawson.
